# Exploring Desmin as a Potential Modifier in Duchenne Muscular Dystrophy–Associated Cardiomyopathy

**DOI:** 10.1111/apha.70117

**Published:** 2025-10-29

**Authors:** Brice‐Emmanuel Guennec, Yeranuhi Hovhannisyan, Gaëlle Revet, Sila Polat, Medhi Hassani, Nathalie Mougenot, Inès Barthelemy, Stephane Blot, Caroline Cieniewski‐Bernard, Arnaud Ferry, Ekaterini Kordeli, Zhenlin Li, Onnik Agbulut

**Affiliations:** ^1^ Institut de Biologie Paris‐Seine (IBPS), UMR CNRS 8263, INSERM U1345, Development, Adaptation and Ageing Sorbonne Université Paris France; ^2^ UMS28, Plateforme d'Expérimentation Cœur, Muscles, Vaisseaux Sorbonne Université Paris France; ^3^ Inserm U955‐E10, IMRB, Université Paris Est, Ecole nationale vétérinaire d'Alfort Maisons‐Alfort France; ^4^ CNRS, UMR 8576—UGSF—Unité de Glycobiologie Structurale et Fonctionnelle Université de Lille Lille France; ^5^ Centre de Recherche en Myologie, UMRS974 Sorbonne Université Paris France; ^6^ Université Pars Cité Paris France

**Keywords:** cardiomyopathy, desmin intermediate filament, disease‐modifier proteins, Duchenne muscular dystrophy, mdx mice

## Abstract

**Aim:**

Duchenne muscular dystrophy (DMD), a rare X‐linked genetic disorder, is affecting skeletal and cardiac muscles due to the loss of the dystrophin protein. Modifier proteins, whose expression is altered in DMD patients, may influence disease progression. Desmin, a muscle‐specific intermediate filament protein, is increased in the skeletal muscle of mdx mice, a murine model of DMD with a mild phenotype. Here, we inquired whether desmin acts as a modifier in DMD‐associated cardiomyopathy.

**Methods:**

Soluble and insoluble desmin levels were quantified in the hearts of two mdx mouse models (B10.mdx and D2.mdx), and GRMD dystrophic dogs. The expression of desmin‐regulatory proteins was also assessed in mdx mice. To assess the impact of desmin levels on the phenotype, we generated mdx mice either desmin‐deficient (mdx‐Des^−/−^) or with reduced levels of desmin by introducing a heterozygous desmin knock‐out allele (mdx‐Des^+/−^). Phenotypic analyses included cardiac function assessment and histological evaluation.

**Results:**

In mdx mice, desmin was elevated in its insoluble, phosphorylated, and presumably filamentous form, while GRMD dogs with a severe DMD‐like phenotype showed no such increase. Desmin deficiency in mdx mice led to severely aggravated dystrophic features, including cardiac dysfunction and increased fibrosis. Moreover, partial desmin reduction in mdx‐Des^+/−^ mice led to the abrogation of insoluble desmin increase and worsened the mild mdx dystrophic phenotype.

**Conclusion:**

Increased filamentous desmin appears to be protective in mdx mouse hearts and may modulate the severity of DMD cardiomyopathy. These findings support a modifier role for desmin and highlight this protein as a potential therapeutic target for DMD.

**Abbreviations**
CASA: chaperone‐assisted selective autophagy
Col1a1: collagen type I alpha 1
Col3a1: collagen type III alpha 1
Ctgf: connective tissue growth factor
DGC: dystrophin‐glycoprotein complex
dKO: double knock‐out
DMD: Duchenne muscular dystrophy
GLS: global longitudinal strain
GRMD: golden retriever muscular dystrophy
h/r: left ventricular wall thickness‐to‐radius ratio
Hmbs: hydroxymethylbilane synthase
Hprt: hypoxanthine phosphoribosyltransferase
HSP: heat shock protein
LTBP4: latent transforming growth factor‐beta binding protein 4
LVEF: left ventricular ejection fraction
LVSF: left ventricular shortening fraction
Myh7: Myosin Heavy Chain 7
Nppa: Natriuretic Peptide A
Nppb: Natriuretic Peptide B
qRT‐PCR: quantitative real‐time PCR
SDS‐PAGE: sodium dodecyl‐sulfate polyacrylamide gel electrophoresis
SEM: standard error of the mean
SNP: single‐nucleotide polymorphism
SPP1: secreted phosphoprotein 1
Sqstm1: sequestosome 1
TGF‐β: transforming growth factor β
Vim: vimentin
WGA: wheat germ agglutinin
WT: wild‐type

## 1 Introduction

Duchenne muscular dystrophy (DMD) is an X‐linked neuromuscular disorder and one of the most severe forms of inherited muscular dystrophies, characterized by progressive skeletal muscle weakness and wasting [1] associated with dilated cardiomyopathy, arrhythmias, and congestive heart failure [2]. DMD is caused by mutations in the *DMD* gene which result in the loss of dystrophin, a large protein of the cortical cytoskeleton with a crucial role in the maintenance of muscle cell integrity [1, 3]. Dystrophin is a core component of the dystrophin‐glycoprotein complex (DGC) [4] at the plasma membrane of skeletal, smooth, and cardiac muscle cells [5, 6]. The complex anchors the actin cytoskeleton to the extracellular matrix, ensuring structural membrane integrity and efficient force transmission during contraction [7, 8]. Loss of dystrophin disrupts protein interactions and destabilizes DGC, leading to sarcolemmal fragility, impaired mechanical coupling, and increased susceptibility to contraction‐induced damage, ultimately contributing to the progressive degeneration of muscle fibers [9]. In the heart, as cardiomyocytes deteriorate, they are replaced by fibrotic or adipose tissue, reducing myocardial contractility and impairing electrical conduction [10]. These changes promote arrhythmias and increase the risk of heart failure [11, 12]. This pathological remodeling of the cardiac tissue is exacerbated by chronic inflammation [13] and multiple secondary cellular factors, including calcium metabolism abnormalities [14], mitochondrial dysfunction [15, 16] and oxidative stress [17, 18].

Due to advancements in respiratory care and multidisciplinary management, cardiovascular disease is now the leading cause of death for DMD patients [19]. Dystrophin deficit manifests as dilated cardiomyopathy, characterized by left ventricular dilation and a reduced ejection fraction. With fibrosis progression, ventricular function further deteriorates, leading to the onset of atrial and ventricular arrhythmias [19]. Although the absence of dystrophin is a common factor in all DMD patients, disease severity varies considerably among individuals. This variability is largely determined by genetic factors, particularly the DMD locus, which is the primary genetic modifier of the disease [20]. The reading frame rule plays a crucial role in genotype–phenotype modulation: when the reading frame is preserved, truncated dystrophin expression is possible, leading to a milder form known as Becker muscular dystrophy. Conversely, an out‐of‐frame mutation results in classic DMD with a nearly complete loss of functional dystrophin. However, some mutations defy this rule [21]. For instance, patients with an out‐of‐frame deletion of exon 45, one of the most frequent mutations, often retain ambulation longer than expected [22]. This phenomenon may be explained by spontaneous alternative splicing, promoting a natural exon 44 skipping event and allowing partial dystrophin production [23]. Beyond the DMD locus, other genes act as trans modifiers of disease progression. Among them, secreted phosphoprotein 1 (SPP1/osteopontin) [24, 25] and latent transforming growth factor‐beta binding protein 4 (LTBP4) [21] play a key role. Osteopontin modulates immune responses and extracellular matrix remodeling, while LTBP4 regulates the bioavailability of transforming growth factor β (TGF‐β), a key factor in muscle fibrosis. Variants in *SPP1* and *LTBP4* genes have been associated with disease severity in DMD patients, with certain polymorphisms correlating with slower progression and prolonged ambulation [26]. To date, most identified genetic modifiers are involved in inflammatory and pro‐fibrotic pathways. However, some notable exceptions have emerged, such as α‐actinin‐3, which modulates sarcomeric function and muscle strength [27, 28, 29].

Desmin is a major component of type III intermediate filaments, specifically expressed in striated, smooth, and cardiac muscle cells [30]. Although it is not part of the DGC, desmin strengthens sarcomeric stability by linking myofibrils to the sarcolemma and by interacting with DGC‐associated proteins [31, 32]. Similar to dystrophin, desmin is essential for maintaining muscle integrity and force transmission. Its role is particularly critical in aligning and anchoring myofibrils at the Z‐discs [33, 34], as well as in organizing and connecting cellular organelles, including the nucleus, mitochondria, and the sarcoplasmic reticulum [35]. Inside cells, desmin exists in both soluble and insoluble forms: the soluble pool mainly comprises unassembled or disassembled monomers and oligomers, while the insoluble fraction corresponds to polymerized, filamentous desmin integrated into the cytoskeletal network. Shifts between these two forms are dynamic and can reflect changes in filament organization and/or stability [34]. Mutations in the desmin gene have been associated with dilated cardiomyopathy [36]. However, the precise role of desmin in heart failure remains unclear. Some studies report excessive filamentous desmin accumulation under mechanical stress conditions, suggesting a potential protective effect [34].

In the mdx murine model, used to study DMD, elevated desmin levels have been detected in skeletal muscle [37]. These findings suggested that endogenous desmin may play a role in disease establishment and/or evolution. However, the exact functions of desmin in cardiomyocytes in this pathological context remain unexplored. To address this question, we compared soluble and insoluble desmin levels in different animal DMD models and found that insoluble desmin levels are increased in the cardiac tissue of mdx mice. This increase was associated with enhanced desmin phosphorylation, upregulation of the chaperone proteins αB‐crystallin and HSP27, as well as the co‐chaperone protein BAG3, reduction of protease calpain‐1 levels, and the preserved filamentous state of desmin. Finally, we deleted or reduced desmin in mdx mice and observed a worsening of the dystrophic phenotype, including the development of cardiomyopathy.

## 2 Materials and Methods

### 2.1 Animal Models

#### 2.1.1 Murine Models

All procedures were performed in accordance with National and European legislations, under the license APAFIS#21554 and #37927 (French Ministry of National Education, Higher Education and Research). To generate a double knockout murine model lacking both dystrophin and desmin, mdx mice (C57BL/10ScSn‐mdx/J) were crossed with desmin knock‐out (Des^−/−^, referred to as DesKO) mice (C57Bl/6) [38], initially producing mdx‐Des^+/−^ mice on a hybrid genetic background (C57Bl/10 × C57Bl/6). After five generations of inbreeding, three groups were obtained: mdx‐Des^−/−^ (dKO), mdx‐Des^+/+^ (mdx), and mdx‐Des^+/−^ (mdx‐Des^+/−^) mice. Genotyping of the mice was performed by standard PCR as previously described [38, 39]. Although dKO mice are viable, they are noticeably smaller than the other genotypes and exhibit a reduced lifespan [37]. Wild‐type (C57Bl/6) mice, obtained from Janvier Labs, were used as a control group. Additionally, a second murine model, the D2.mdx (DBA/2J) strain originally obtained from Jackson Laboratory, was used and compared with DBA/2J control mice.

#### 2.1.2 Canine Model

Left ventricular free wall biopsies were collected from Golden Retriever Muscular Dystrophy (GRMD) dogs (*n* = 6) during necropsy, as well as from control dogs (*n* = 3), frozen in isopentane cooled in liquid nitrogen and stored at −80°C. No dog received any treatment except medications included in their veterinary routine follow‐up. Biopsies from GRMD dogs were either taken from young dogs (6 months of age, *n* = 3) or from adult dogs (36 months of age, *n* = 3). The three healthy golden retriever dogs used as controls were aged 7, 14, and 25 months, respectively, at the time of sampling. All these samplings were performed in the context of projects approved by the ethical committee of EnvA, ANSES, and UPEC under the approval numbers 20/12/12‐18 and 2023‐02‐14‐04 (APAFiS #40445).

### 2.2 Extraction of Soluble and Insoluble Protein Fractions From Heart Samples

Fractionation of protein samples was performed using the FOCUS Soluble & Insoluble Protein Extraction Kit (G‐Biosciences). Ventricular tissues were ground in liquid nitrogen using a mortar and pestle to obtain a fine powder for protein extraction. The tissue powder was resuspended in Soluble Protein Extraction buffer, supplemented with phosphatases (Sigma) and proteases (Thermo Fisher Scientific) inhibitors. The suspension was subjected to sonication on ice to disrupt the cells and fragment genomic DNA. The homogenate was then centrifuged at 20 000 *g* for 30 min at 4°C. The supernatant corresponded to the soluble protein fraction and was carefully collected. The pellet was resuspended in 0.3 mL of FOCUS Protein Solubilization buffer and centrifuged again under identical conditions to obtain the insoluble protein fraction. The purity of the extracted fractions was assessed by sodium dodecyl‐sulfate polyacrylamide gel electrophoresis (SDS‐PAGE) and Western blot analysis, using antibodies against sarcomeric myosin (#MF20, Developmental Studies Hybridoma Bank) and myoglobin (#NBPS‐67599, NovusBio) as markers for insoluble and soluble protein fractions, respectively (Figure S1). For total protein extraction, the tissue powder was resuspended in Newcastle buffer (75 mM Tris, pH 6.8, 3.8% SDS, 4 M urea, and 20% glycerol) [40], supplemented with phosphatase and protease inhibitors. The suspension was incubated on ice for 30 min with continuous agitation. Proteins were quantified using the Bradford colorimetric assay (BioRad) for soluble and insoluble fractions, and the Pierce BCA Protein Assay Kit (Thermo Fisher Scientific) for total protein extracts.

### 2.3 Electrophoretic Separation of Proteins and Western‐Blot

#### 2.3.1 SDS‐Page

Following 5 min incubation at 95°C, 5 μg of proteins in 1X Laemmli buffer (BioRad) were loaded onto 10% acrylamide Tris‐Glycine gels (Novex WedgeWell 1.0 mm, Thermo Fisher Scientific) and migrated at 100 V for 2 h using the Mini Gel Tank system and migration buffer (190 mM glycine, 25 mM Tris base, and 0.1% SDS) (Thermo Fisher Scientific).

#### 2.3.2 Phos‐Tag PAGE

Five μg of protein was loaded onto a 1.5 mm thick Phos‐tag gel. The gel consisted of a 7.5% and 12% acrylamide separation gel for desmin and αB‐crystallin respectively, prepared as previously described [41]. Electrophoresis was performed under a constant current of 25 mA per gel in the migration buffer for 150 min using the Mini‐PROTEAN gel system (BioRad). Gels were then rinsed twice for 7 min in a transfer buffer (BioRad) containing 1 mM EDTA, followed by two 7 min rinses in transfer buffer without EDTA.

#### 2.3.3 Wheat Germ Agglutinin‐PAGE

Five μg of protein was loaded onto a 1 mm thick Wheat Germ Agglutinin (WGA) gel, consisting of a 7.5% acrylamide separation gel for desmin and a 12.5% acrylamide separation gel for αB‐crystallin, prepared as previously described [41]. Electrophoresis was performed under a constant current of 25 mA per gel in the migration buffer (190 mM Glycine, 25 mM Tris base, 0.1% SDS) for 100 min using the mini‐protean gel system (BioRad).

#### 2.3.4 Western Blot

Following electrophoresis, proteins separated by SDS‐PAGE, Phos‐tag PAGE, or WGA‐PAGE were transferred onto 0.2 μm PVDF or nitrocellulose membranes using the i‐Blot 3 transfer system (Thermo Fisher Scientific) at 25 V for 10 min with cooling. To visualize the total protein content, membranes were incubated for 10 min in No‐Stain solution (Thermo Fisher Scientific) protected from light. After incubation, the membranes were rinsed with water three times for 2 min and imaged using the ChemiDoc MP system (BioRad) with the Sypro Ruby Red program. The membranes were blocked with 5% non‐fat dry milk in PBS with 0.01% Tween‐20 (PBS‐T). The membranes were incubated overnight at 4°C with gentle agitation in blocking solution containing the following primary antibodies: Desmin (#ab32362, 1:1000, Abcam), αB‐crystallin (#ab11061, 1:50 000, Abcam), HSP27 (#PA5‐85351, 1:1000, Thermo Fisher Scientific), Calpain‐1 (#2556s, 1:1000, Cell Signaling), and BAG3 (#10599‐1‐AP, 1:5000, Proteintech). After three 10 min washes in PBS‐T, the membranes were incubated in blocking solution with secondary antibodies (HRP‐linked IgG, #A0545 or #A4416, Sigma) for 1 h at room temperature. Following three 5 min washes in PBS‐T, positive protein bands were detected by chemiluminescence using ECL SuperSignal West Pico PLUS (Thermo Fisher Scientific) for SDS‐PAGE and Phos‐tag PAGE, and ECL SuperSignal West Femto Plus (Thermo Fisher Scientific) for WGA‐PAGE and visualized with the ChemiDoc MP system (BioRad). Quantification of the protein bands was performed by optical densitometry and analyzed using the FIJI digital image processing software. The expression of each analyzed protein band was normalized to the total lane signal obtained with No‐Stain. Full‐length images of PVDF membranes are shown in Figures S2–S5.

### 2.4 Quantitative Real‐Time PCR

Total RNA was extracted from cardiac tissue using TRIzol reagent (Thermo Fisher Scientific), according to the manufacturer's instructions, and quantified using a NanoDrop (Thermo Fisher Scientific). A total of 1 μg of RNA was used to synthesize the first‐strand cDNA using the RevertAid First Strand cDNA Synthesis Kit (Thermo Fisher Scientific) with random hexamers, according to the manufacturer's protocol. Gene expression was quantified by quantitative real‐time PCR (qRT‐PCR) using the CFX Opus 384 Real‐Time PCR System (BioRad). Each sample was run in triplicate in a 6 μL reaction volume containing 3 μL of SYBR Green Master Mix and 3 μL of diluted cDNA (1:25) containing 500 nM of each forward and reverse primer. The thermal cycling profile for qRT‐PCR was as follows: 95°C for 8 min, followed by 35 cycles of 15 s denaturation at 95°C, 15 s annealing at 60°C, and 30 s extension at 72°C. To prevent amplification of genomic DNA, primers were designed, when possible, to span exon–exon junctions. Two reference genes, *Hmbs* (Hydroxymethylbilane synthase) and *Hprt* (Hypoxanthine phosphoribosyltransferase), were used for data normalization. Data collection and analysis were performed using qRT‐PCR Analysis Software (BioRad).

### 2.5 Histological and Immunohistochemical Staining

For histological and immunohistochemical analysis, the atria were separated from the ventricles. Ventricles were then bisected along the short axis through the central part of the heart. Both halves were embedded in OCT (Thermo Fisher Scientific) and immediately frozen in liquid isopentane cooled with liquid nitrogen. Cardiac sections of 7 μm were obtained using a cryostat (Thermo Fisher Scientific).

For immunofluorescence, unfixed frozen sections were saturated for 1 h in blocking solution (PBS containing 5% bovine serum albumin), then incubated for 1 h with primary antibodies against desmin (#ab32362, Abcam and #M0760, DAKO), α‐actinin (#A7811, Sigma), and desmoplakin (#MA1‐83118, Thermo Fisher Scientific) in PBS containing 2.5% bovine serum albumin. After washing with PBS, the sections were incubated for 1 h with fluorochrome‐coupled secondary antibodies (Alexa Fluor, Invitrogen). The nuclei were stained with Hoechst (BD Biosciences) for 5 min. Finally, the sections were mounted with Mowiol (Sigma‐Aldrich). Images were captured using a Leica LMS 980 confocal inverted microscope.

To visualize fibrosis, frozen sections were stained with Sirius Red (Sigma‐Aldrich), mounted in Eukitt (Sigma‐Aldrich), and examined by optical microscopy. Images were captured using a Leica DMi8 microscope equipped with a digital camera.

### 2.6 Transmission Electron Microscopy

To assess the ultrastructural integrity of cardiomyocytes, cardiac tissue samples were fixed overnight at 4°C in a solution of 2% glutaraldehyde and 2% paraformaldehyde in 0.2 M phosphate buffer (pH 7.4), followed by post‐fixation with 1% osmium tetroxide [42]. After dehydration in a series of increasing ethanol and acetone baths, the samples were embedded in epoxy resin. Ultrathin sections (70 nm) were cut using an ultramicrotome (Leica UC6), placed on copper and rhodium‐coated grids, and contrasted with UranyLess (Delta Microscopies) solution and 0.2% lead citrate. Observations were performed using a transmission electron microscope (Zeiss 912 Omega) operating at 80 kV, equipped with a digital camera (Emsis Veleta 2kx2k).

### 2.7 Echocardiography

Measurements of left ventricular dimensions and volumes were performed by transthoracic echocardiography using the VEVO 3100 Imaging system (FUJIFILM VisualSonics, Netherlands) with an ultrasound probe MX550D from a 25 to 55 MHz frequency range. For these assessments, mice were anesthetized under isoflurane (induction with 2% and maintenance with 0.5%). All measurements were performed on digital loops in triplicate and used the M‐mode corrected cube formula. Using Vevo LAB software, data were analyzed blindly to the treatment group.

### 2.8 Statistical Analysis

All experimental data are presented as mean ± standard error of the mean (SEM). Normality was checked using the Shapiro–Wilk test and, if necessary, non‐parametric tests were used. The unpaired Student's *t*‐test was used for comparisons between two groups if normality was respected; otherwise, a Mann–Whitney test was used. When multiple comparisons were required, a one‐way ANOVA test combined with Dunnett's multiple comparison test was used if normality was respected; otherwise, a Kruskal–Wallis test combined with Dunn's multiple comparison test was used. The presence of outliers in each dataset was evaluated using Grubbs' test, and identified outliers were removed if statistically justified (*p* < 0.05). *p* values of less than 0.05 (*), 0.01 (**), 0.001 (***) and 0.0001 (****) were considered statistically significant. Data were analyzed and presented using Prism v.10.4.2 (GraphPad).

## 3 Results

### 3.1 Filamentous Desmin Accumulates in the Heart of mdx Mice

**FIGURE 1:**
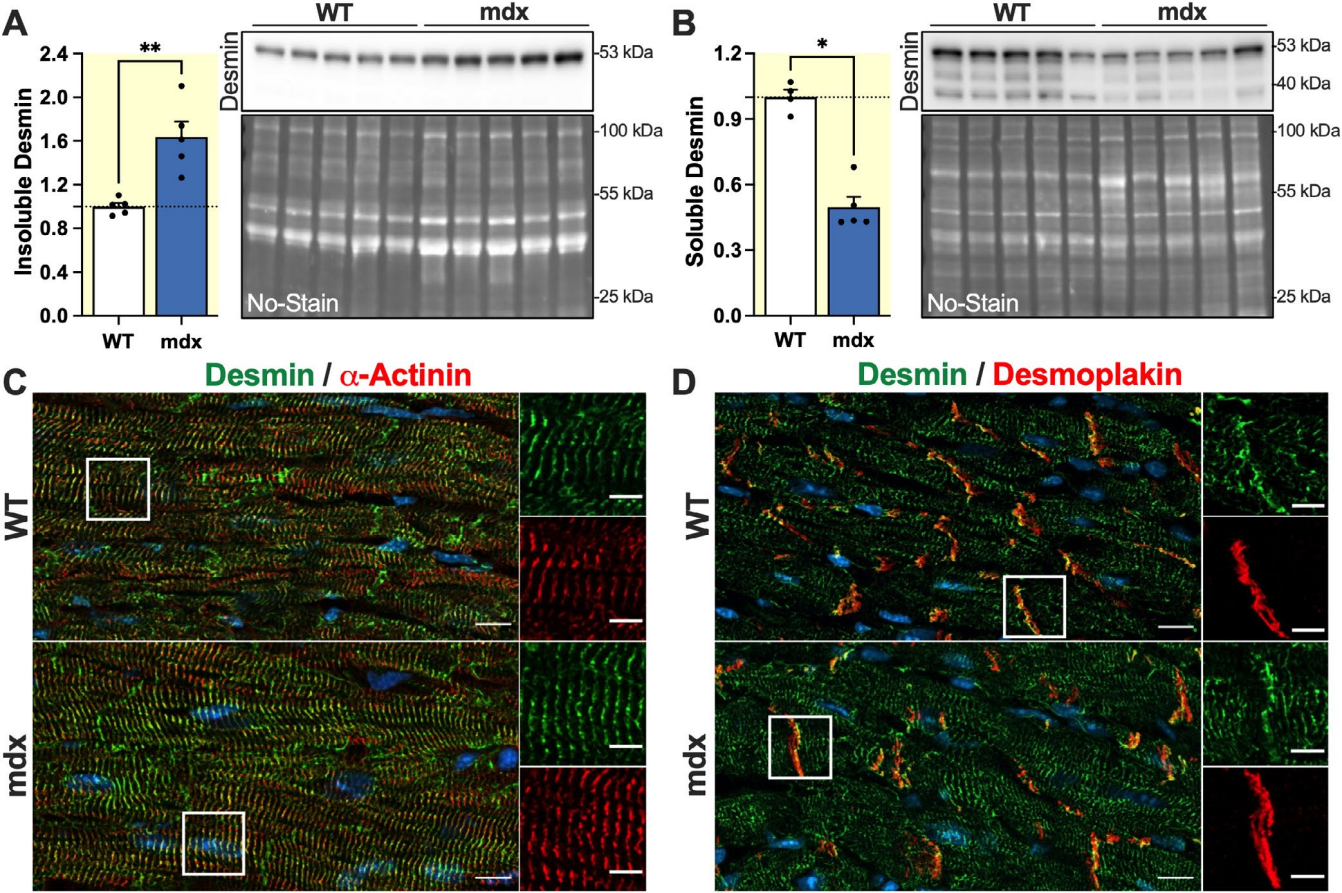
Increased filamentous desmin in mdx hearts. (A, B) Western blots and corresponding densitometry analysis of desmin insoluble (A) and soluble (B) protein fractions extracted from hearts of 4‐month‐old male WT and mdx mice (*n* = 5 per group). Protein levels were normalized to total protein band profile (No‐Stain) per lane. The bands lower than 53 kDa in the soluble fraction may represent products of proteolytic cleavage or alternatively spliced isoforms of desmin. A Grubbs' test was used to exclude one outlier in the WT group for the soluble desmin fraction. Data are expressed as mean values ±SEM. *p < 0.05, **p < 0.01. Full length images of PVDF membranes are shown in Figure S2. (C, D) Representative immunofluorescence images of frozen ventricular sections from 4‐month‐old male WT and mdx mice. Desmin (green) was co‐stained with either α‐actinin (red, C) or desmoplakin (red, D). Nuclei were counterstained with Hoechst (blue). Images on the left show low‐magnification (Scale bars: 10 μm) views of sarcomeres (C) and intercalated discs (D). Selected regions indicated by rectangles are shown at higher magnification (Scale bars: 5 μm) on the right.

To find out whether dystrophin loss affects the expression and/or state of assembly of desmin in the heart, we first measured desmin mRNA levels by qRT‐PCR and found no significant difference between mdx and wild‐type (WT) mice (56.05 ± 3.90 in mdx vs. 50.16 ± 1.47 in WT, *p* > 0.05). We then investigated total desmin protein levels and partitioning between soluble and insoluble fractions in mdx and WT mice, following differential protein extraction. Western blot analysis showed no significant difference in total desmin protein levels (Figure S6). However, a significant increase was found in insoluble desmin in mdx hearts compared to WT controls (1.64 ± 0.14‐fold, *p* < 0.01) (Figure 1A), along with a reduction in the soluble fraction (0.50 ± 0.05‐fold, *p* < 0.05) (Figure 1B). These changes could indicate potential alterations in desmin assembly and subcellular localization in cardiomyocytes. We performed desmin immunolabeling on frozen heart sections from mdx and WT mice, focusing on two key structures of cardiomyocytes where desmin accumulates: the sarcomeres and the intercalated discs, as identified by α‐actinin and desmoplakin labeling, respectively. Confocal image analysis revealed that filamentous desmin maintains normal organization and striation in both mdx and WT mice, as indicated by Z‐line pattern and intercalated disc structure (Figure 1C,D). Moreover, desmin protein aggregates were not detected in these cells. These findings suggest that the accumulation of insoluble desmin in mdx cardiomyocytes primarily corresponds to an increase in the filamentous network, indicating a potential compensatory mechanism in mdx mice.

### 3.2 Desmin Protein Appears to Be Stabilized in the Heart of mdx Mice

**FIGURE 2:**
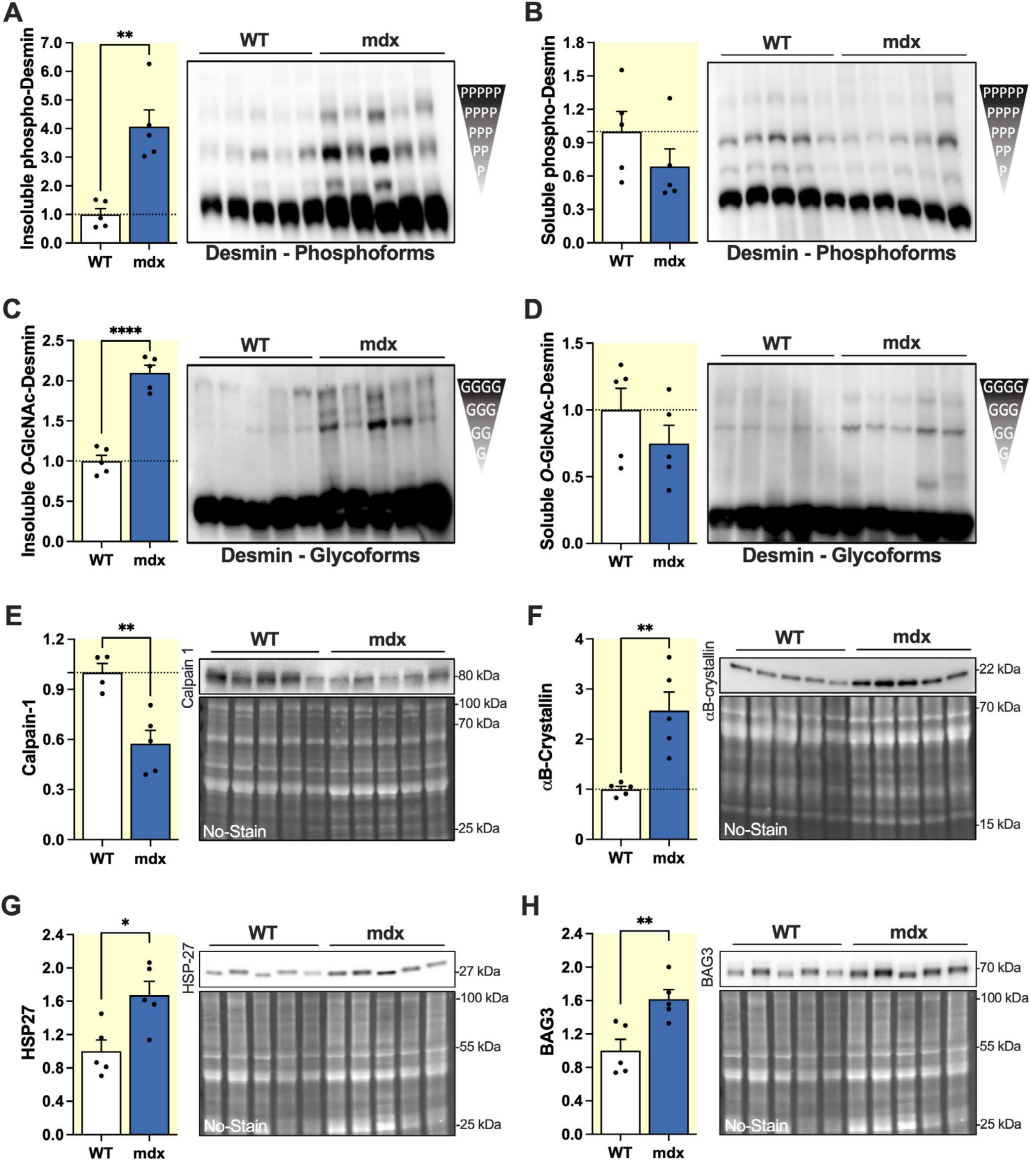
Post‐translational modifications of desmin and expression of filament stability‐related proteins in WT and mdx hearts. (A, B) Western blots following Phos‐tag PAGE and corresponding densitometric analyses of the phosphorylation state of insoluble (A) and soluble (B) desmin. (C, D) Western blots following WGA‐PAGE and corresponding densitometric analyses of changes in *O*‐GlcNAcylation of insoluble (C) and soluble (D) desmin. Total phosphorylation and *O*‐GlcNAcylation levels were calculated by summing the intensity of all retarded desmin bands and normalizing to the total protein band profile per lane (No‐Stain). (E–H) Western blots and corresponding densitometry analysis of Calpain‐1 (E), αB‐crystallin (F), HSP27 (G), and BAG3 (H) protein levels. Protein quantification was normalized to the total protein band profile per lane (No‐Stain). A Grubbs' test was used to exclude one outlier in the WT group for calpain‐1. Results are expressed as mean values ±SEM. Heart extracts from 4‐month‐old male WT and mdx mice (*n* = 5 per group) were used for all analyses. *p < 0.05, **p < 0.01, ****p < 0.0001. Full‐length images of PVDF membranes are shown in Figure S3.

One explanation for increased levels of filamentous desmin could be enhanced stability. It is known that desmin undergoes various post‐translational modifications which can be implicated in filament assembly and stability [43, 44]. We investigated two major modifications, phosphorylation and *O*‐GlcNAcylation. Due to the presence of multiple known phosphorylation sites on desmin and the lack of specific antibodies for many of them, we opted for a global approach to study phosphorylation. The Phos‐tag PAGE technique was used to detect differently migrating phosphorylated desmin polypeptides in each sample. In this electrophoretic approach, the migration of phosphorylated proteins is retarded in a standard SDS‐PAGE system due to the presence of a phosphate‐binding molecule co‐polymerized with the acrylamide gel. Following electrophoresis, phosphorylated desmin was determined by Western blot. Each positive band corresponded to a different phosphorylated form of desmin, with upper bands representing more heavily phosphorylated forms, and the major lower band corresponding to the non‐phosphorylated forms. Phosphorylation of desmin was increased by 4.08 ± 0.58‐fold in the insoluble mdx fraction (*p* < 0.01) compared to WT (Figure 2A). By normalizing phosphorylated desmin in two steps—first to the total protein loading, then to the total desmin levels for each sample as shown in Figure 1—we ensured that the observed increase in phosphorylation was not simply due to increased total desmin levels in the insoluble mdx fraction but rather reflected a specific modification of desmin in mdx tissue (2.67 ± 0.51‐fold in the insoluble fraction, *p* < 0.05, Figure S7). Furthermore, the increase in phosphorylated protein levels was consistent across the different desmin bands, suggesting a global modification of this protein. In contrast, no significant changes were observed in the soluble fraction (Figure 2B), indicating that this modification is specific to insoluble desmin.

By a similar approach, we analyzed desmin *O*‐GlcNAcylation using the WGA‐PAGE method, which detects *O*‐GlcNAc‐modified proteins by lectin affinity gel electrophoresis. At first sight, *O*‐GlcNAcylation appeared increased by 2.10 ± 0.09‐fold in the insoluble mdx fraction of desmin (*p* < 0.0001) compared to WT (Figure 2C), whereas no significant differences were observed in the soluble fraction (Figure 2D). However, following the two‐step normalization, no significant differences in *O*‐GlcNAc‐modified desmin were observed between mdx and WT in either soluble or insoluble desmin fractions (Figure S7), suggesting that the loss of dystrophin does not induce a major effect on desmin *O*‐GlcNAcylation.

In addition to desmin modifications, we further examined other proteins known to regulate desmin filament turnover and integrity. We focused on calpain‐1, a protease involved in desmin filament disassembly; αB‐crystallin and HSP27, both chaperone proteins essential for proper intermediate filament folding and protection against stress; and BAG3, a co‐chaperone involved in protein quality control and turnover. Our results showed a significant decrease in calpain‐1 levels (0.58 ± 0.08‐fold, *p* < 0.01) in mdx hearts compared to WT (Figure 2E). This decrease suggests reduced filament degradation. Additionally, both αB‐crystallin (2.57 ± 0.37‐fold, *p* < 0.01) and HSP27 (1.67 ± 0.17‐fold, *p* < 0.05) levels were significantly increased in mdx mice (Figure 2F,G). BAG3 expression was also elevated (1.62 ± 0.11‐fold, *p* < 0.01) in mdx hearts compared to WT (Figure 2H).

The increase in desmin filament phosphorylation, together with decreased calpain‐1 levels and elevated expression of αB‐crystallin, HSP27, and BAG3, points to changes in the regulation of desmin filament stability under conditions of dystrophin loss.

### 3.3 Increase of Insoluble Desmin Correlates With an Attenuated Dystrophic Phenotype

**FIGURE 3:**
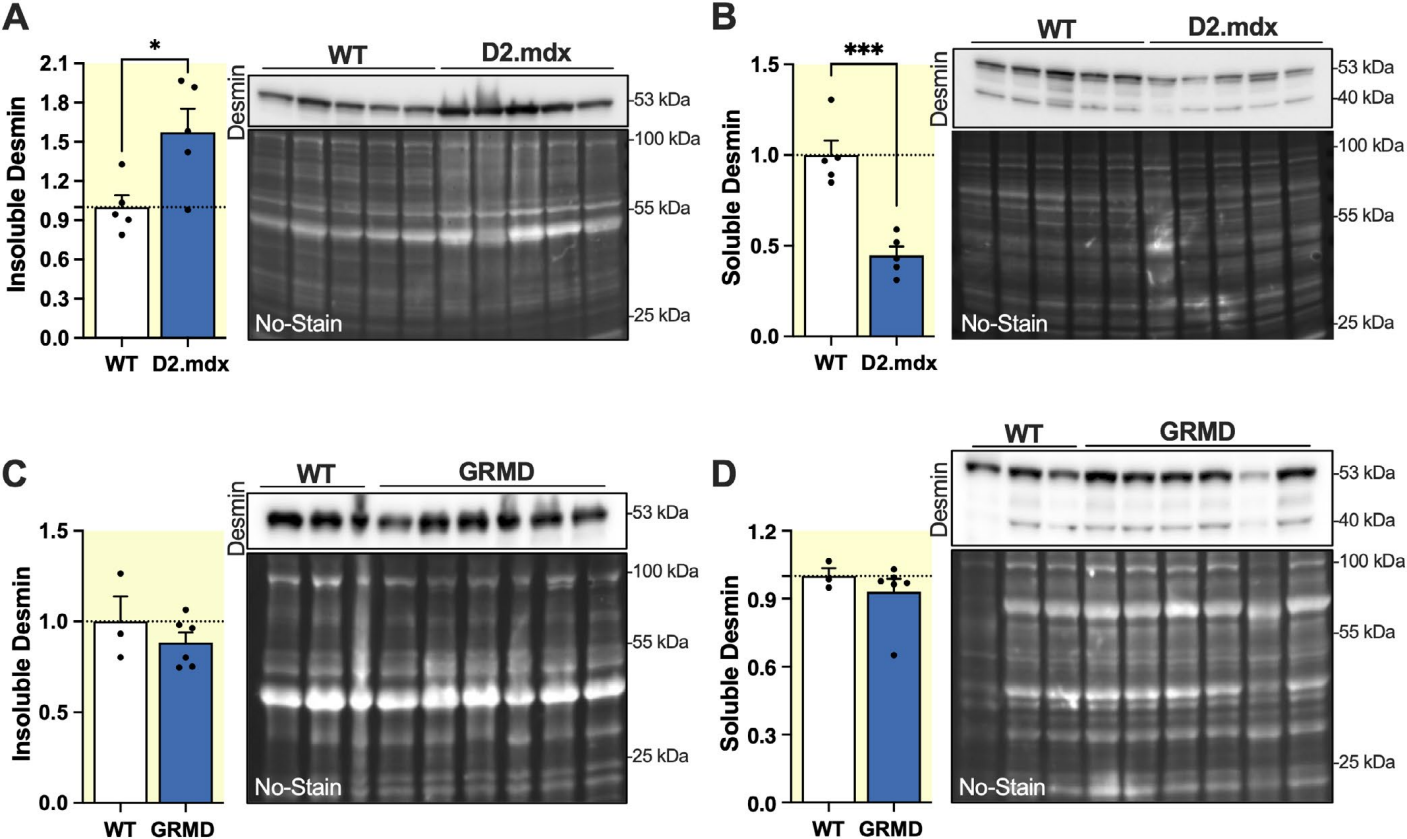
Elevated levels of insoluble desmin correlate with reduced severity of the dystrophic phenotype. (A–D) Western blots and corresponding densitometric analyses of desmin levels in insoluble (A, C) and soluble (B, D) protein fractions extracted from hearts of 4‐month‐old male WT and D2.mdxd mice (A, B) (*n* = 5 per group), and from the left ventricle of WT (*n* = 3) and GRMD (*n* = 6) dogs aged 6 to 36 months (C, D). Protein levels were normalized to the total protein band profile (No‐Stain) per lane and are expressed as mean values ±SEM. *p < 0.05, ***p < 0.001. Full length images of PVDF membranes are shown in Figure S4.

To determine whether the observed increase in insoluble desmin is directly associated with the pathological condition, we compared findings from mdx mice with two additional animal models of DMD: the D2.mdx mouse (DBA/2J strain), which exhibits a similar cardiac phenotype to that of mdx mice [45, 46], and the GRMD (Golden Retriever Muscular Dystrophy) canine model, which presents a DMD‐like phenotype [47, 48]. Quantitative analysis of desmin by Western blot in D2.mdx mice revealed a significant increase in the insoluble fraction (1.57 ± 0.18‐fold, *p* < 0.05) and a decrease in the soluble fraction (0.45 ± 0.05‐fold, *p* < 0.001) compared to WT (Figure 3A,B), in agreement with mdx mice. In contrast, no significant changes in desmin levels were observed in GRMD dogs, in both soluble and insoluble fractions, compared to WT dogs (Figure 3C,D). These findings suggest that further accumulation of insoluble desmin may be associated with less severe or milder phenotypes of muscular dystrophy and may play a role in disease progression.

### 3.4 Deletion of Desmin Leads to Aggravation of the mdx Cardiac Phenotype

**FIGURE 4:**
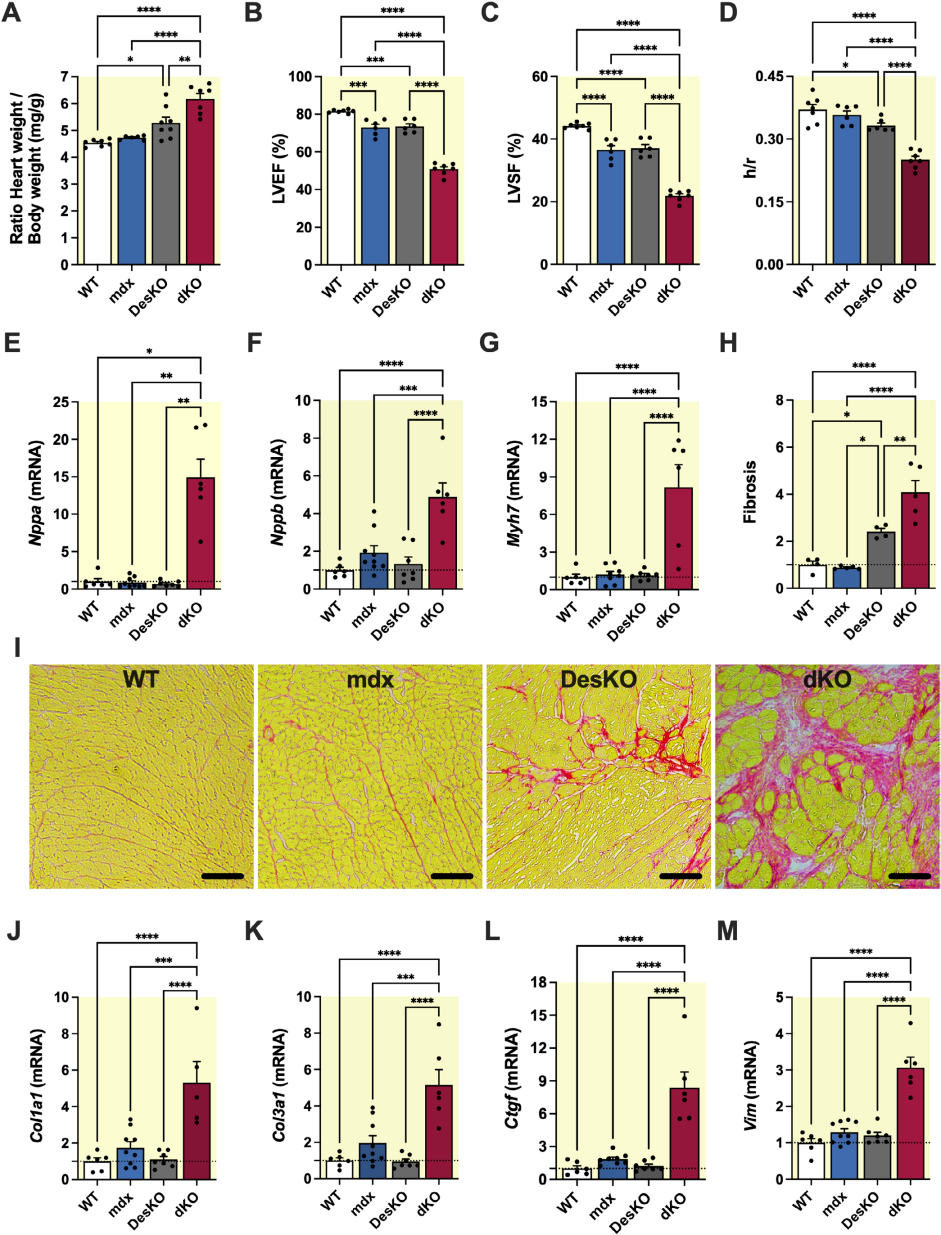
Desmin deletion exacerbates the dystrophic phenotype in mdx mice. (A–D) Morphological and echocardiographic parameters measured in 2‐month‐old WT, mdx, DesKO and mdx‐Des^−/−^ (dKO) male mice (*n* = 6–7). (A) Heart weight to body weight ratio. (B) Left ventricular ejection fraction (LVEF). (C) Left ventricular shortening fraction (LVSF). (D) Left ventricular wall thickness‐to‐radius ratio (h/r). (E–G) Quantification of mRNA levels of cardiac stress and remodeling markers (*Nppa*, *Nppb* and *Myh7*) by qRT‐PCR in 4‐month‐old WT, mdx, DesKO and dKO male mice (*n* = 6–8). (H, I) Assessment of cardiac fibrosis in 2‐month‐old WT, mdx, DesKO and dKO male mice (*n* = 4–5). (H) Quantification of collagen deposition using Sirius Red staining. (I) Representative histological images of Sirius Red–stained cardiac sections. Scale bars: 100 μm. (J–M) Quantification of mRNA levels of fibrosis‐related gene expression (*Col1a1*, *Col3a1*, *Ctgf*, *Vim*) in heart tissue from 4‐month‐old WT, mdx, DesKO and dKO male mice (*n* = 6–8). Results are expressed as mean values ±SEM. *p < 0.05, **p < 0.01, ***p < 0.001, ****p < 0.0001.

To further support a role for desmin in the manifestation of the dystrophic phenotype, we studied the effect of desmin ablation in mdx mice (dKO: mdx‐Des^−/−^ mice). We observed significant alterations in cardiac morphological and functional parameters. Morphologically, mice lacking desmin (DesKO) exhibited a significant increase in the heart weight‐to‐body weight ratio compared to WT (1.16 ± 0.05‐fold, *p* < 0.05), indicating early cardiac remodeling. This increase was even more pronounced in dKO mice (1.36 ± 0.04‐fold, *p* < 0.0001) (Figure 4A), consistent with exacerbated remodeling. Echocardiographic assessment revealed significant impairment in cardiac function. When compared to WT, both left ventricular ejection fraction (LVEF) and left ventricular shortening fraction (LVSF) were significantly reduced in mdx (0.90 ± 0.02‐fold, *p* < 0.001 for LVEF and 0.83 ± 0.03‐fold, *p* < 0.0001 for LVSF) and DesKO mice (0.90 ± 0.02‐fold, *p* < 0.001 for LVEF and 0.84 ± 0.03‐fold, *p* < 0.0001 for LVSF) (Figure 4B,C). This reduction was even more pronounced in dKO mice (0.62 ± 0.01‐fold, *p* < 0.0001 for LVEF and 0.50 ± 0.01‐fold, *p* < 0.0001 for LVSF), highlighting further deterioration of the cardiac function in the absence of desmin. In parallel, a significant decrease in the left ventricular wall thickness‐to‐radius ratio (h/r) in dKO mice compared to WT mice (0.68 ± 0.02‐fold, *p* < 0.0001) indicated a relative thinning of the left ventricular wall (Figure 4D). These morphological and functional changes correlated with an increased expression of cardiac stress and remodeling markers. Notably, the gene expression levels of Natriuretic Peptide A (*Nppa*), Natriuretic Peptide B (*Nppb*), and Myosin Heavy Chain 7 (Myh7) were significantly elevated in the heart of dKO mice compared to WT mice (14.92 ± 2.43‐fold, *p* < 0.05; 4.88 ± 0.74‐fold, *p* < 0.0001; 8.17 ± 1.80‐fold, *p* < 0.0001, respectively), to mdx mice (17.11 ± 2.79‐fold, *p* < 0.01; 2.53 ± 0.38‐fold, *p* < 0.001; 6.73 ± 1.49‐fold, *p* < 0.0001, respectively), and to DesKO mice (21.73 ± 3.54‐fold, *p* < 0.01; 3.67 ± 0.56‐fold, *p* < 0.0001; 7.15 ± 1.58‐fold, *p* < 0.0001, respectively) (Figure 4E–G).

Histological analysis further assessed the impact of desmin loss on cardiac fibrosis in mdx mice. When compared to WT, a significant increase in fibrosis was observed in dKO hearts (4.08 ± 0.50‐fold, *p* < 0.0001), mdx (4.64 ± 0.57‐fold, *p* < 0.0001), and DesKO (1.69 ± 0.21‐fold, *p* < 0.01) mice, as demonstrated by Sirius Red staining (Figure 4H,I). This excessive fibrotic accumulation was corroborated by a marked upregulation of fibrosis‐related gene expression in dKO hearts, including collagen type I alpha 1 (*Col1a1*), collagen type III alpha 1 (*Col3a1*), connective tissue growth factor (*Ctgf*), and vimentin (*Vim*) (Figure 4J–M).

**FIGURE 5:**
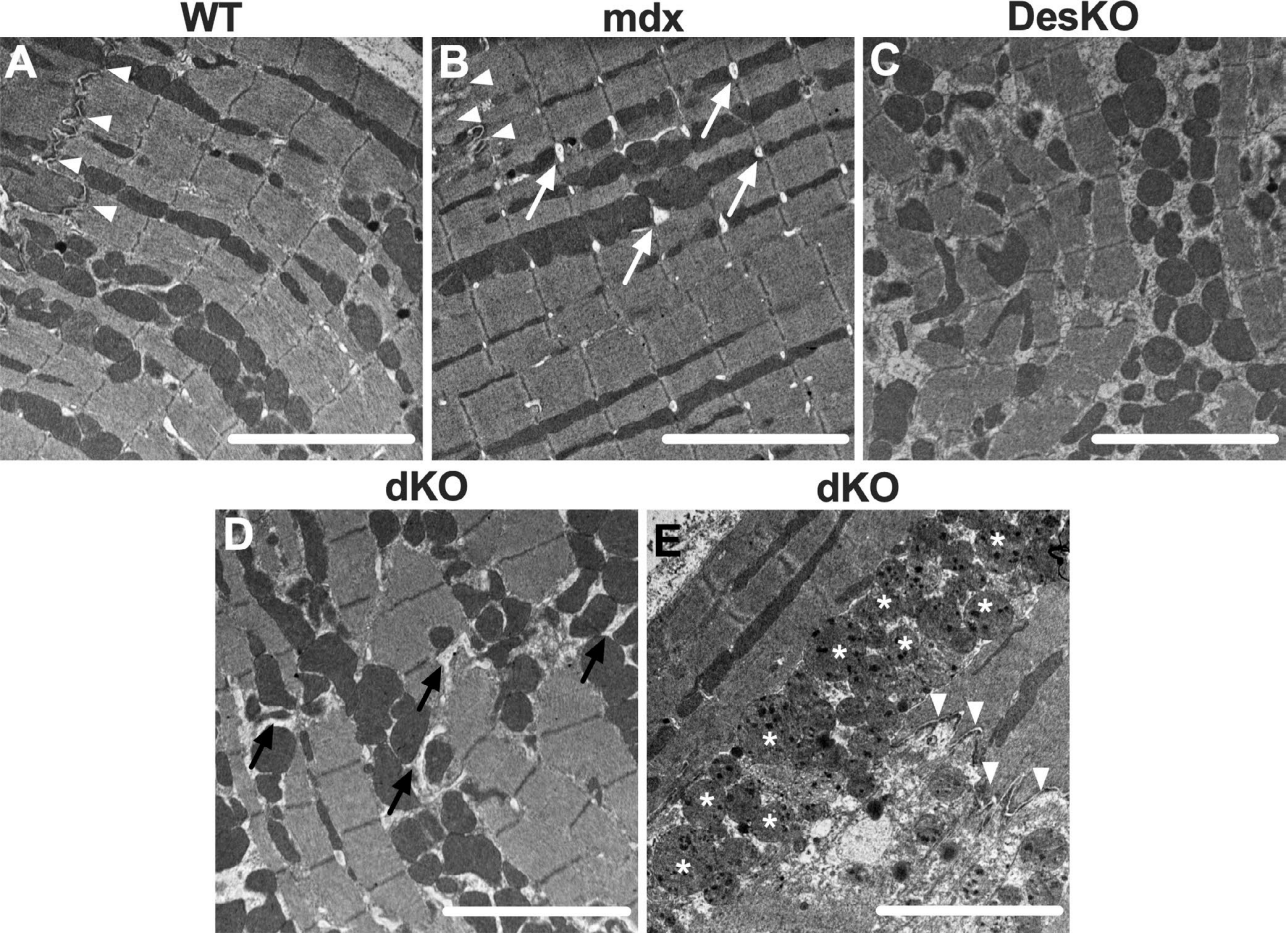
Cardiomyocyte morphology is severely disrupted in the left ventricle of dKO mice. Transmission electron microscopy images of ultrathin sections from the left ventricular myocardium of 2‐month‐old male (A) WT, (B) mdx, (C) DesKO and (D, E) dKO mice. Asterisks (*) indicate swollen mitochondria; white arrows indicate swollen T‐tubules; black arrows indicate swollen sarcoplasmic reticulum and white arrowheads point to intercalated discs. Scale bar = 1 μm.

To further investigate the consequences of desmin ablation at the tissue and cell level, we performed ultrastructural analysis of left ventricular myocardium from 2‐month‐old male mice using electron microscopy. Structural integrity appears preserved in both mdx and WT cardiomyocytes, with only minor alterations in mdx, such as swollen T‐tubules (Figure 5A,B). In DesKO heart tissue (Figure 5C), moderate structural alterations were observed, including misaligned muscle fibers and Z‐lines, partial sarcomeric disorganization, and localized mitochondrial clustering. In contrast, cardiomyocytes in dKO hearts displayed mitochondrial alterations, severe sarcomeric disorganization and misalignment, and swollen sarcoplasmic reticulum between mitochondria and sarcomeres (Figure 5D,E). Figure 5E shows locally disintegrated cardiomyocytes separated by a deformed intercalated disc, with loss of sarcomeric structure and accumulation of swollen and severely damaged mitochondria. Overall, our findings demonstrate that desmin ablation in mdx mice exacerbates cardiac alterations by affecting tissue organization, cell morphology, and function. The loss of desmin thus leads to aggravation of the dystrophic phenotype, further compromising cardiac function in mdx mice.

### 3.5 Abrogation of the Increase of Insoluble Desmin Levels Aggravates the Dystrophic Phenotype in mdx Mice

**FIGURE 6:**
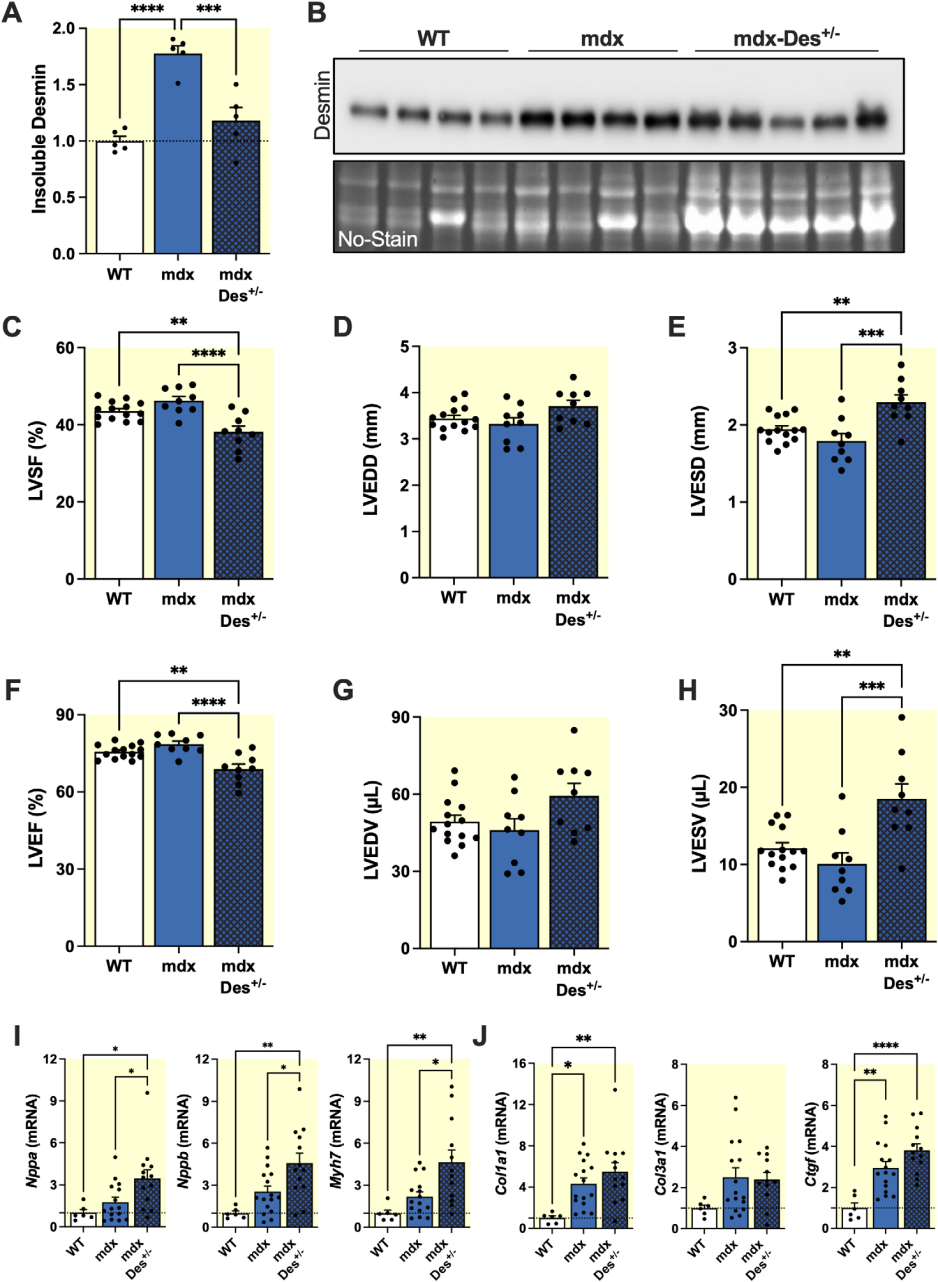
Restoration of WT levels of desmin in mdx hearts leads to aggravation of the mdx dystrophic phenotype. (A, B) Western blots and corresponding densitometric analyses of desmin in the insoluble fraction extracted from hearts of 4‐month‐old male WT, mdx, and mdx‐Des^+/−^ mice (*n* = 4–5 per group). Protein levels were normalized to the total protein band profile (No‐Stain) per lane. Full‐length images of PVDF membranes are shown in Figure S5. (C–H) Cardiac function was evaluated by echocardiography in 6‐month‐old WT, mdx, and mdx‐Des^+/−^ mice. Parameters include: Left ventricular shortening fraction (LVSF) (C), Left ventricular end‐diastolic diameter (LVEDD) (D), Left ventricular end‐systolic diameter (LVESD) (E), Left ventricular ejection fraction (LVEF) (F), Left ventricular end‐diastolic volume (LVEDV) (G), and Left ventricular end‐systolic volume (LVESV) (H). (I, J) Quantification of mRNA levels of cardiac stress and remodeling markers (*Nppa*, *Nppb*, *Myh7*) (I) and fibrosis markers (*Col1a1*, *Col3a1*, *Ctgf*) (J) by qRT‐PCR in 6‐month‐old WT, mdx, and mdx‐Des^+/−^ mice (*n* = 13–16). Results are expressed as mean values ±SEM. *p < 0.05, **p < 0.01, ***p < 0.001, ****p < 0.0001.

We crossed mdx mice with desmin knockout mice, generating offspring (mdx‐Des^+/−^) with a single functional *Des* allele. In the hearts of these mice, desmin mRNA levels were reduced by 0.42 ± 0.06‐fold (*p* < 0.001) compared to mdx mice. This genetic approach allowed us to investigate the impact of partial desmin reduction in the context of dystrophin deficiency. Quantitative analysis of desmin by Western blot in mdx‐Des^+/−^ mice revealed a significant decrease in the insoluble fraction (0.66 ± 0.07‐fold, *p* < 0.001) compared to mdx mice, with levels not significantly different from WT controls (*p* > 0.05) (Figure 6A,B). Echocardiographic assessment revealed a more pronounced systolic dysfunction in mdx‐Des^+/−^ mice compared to mdx and WT mice (Figure 6C–H). Compared to mdx mice, both the LVSF (Figure 6C) and LVEF (Figure 6F) were significantly reduced in mdx‐Des^+/−^ mice (46.22% ± 1.11% in mdx vs. 38.16% ± 1.50% in mdx‐Des^+/−^ mice for LVSF, *p* < 0.0001; 78.53% ± 1.20% in mdx vs. 68.92% ± 1.90% in mdx‐Des^+/−^ mice for LVEF, *p* < 0.0001). This functional decline was accompanied by an increase in left ventricular end‐systolic diameter (LVESD) (1.79 ± 0.10 mm in mdx vs. 2.29 ± 0.10 mm in mdx‐Des^+/−^ mice, *p* < 0.001, Figure 6E), left ventricular end‐systolic volume (LVESV) (10.08 ± 1.43 μL in mdx vs. 18.51 ± 1.93 μL in mdx‐Des^+/−^ mice, *p* < 0.001, Figure 6H), indicating ventricular dilation and adverse remodeling. In contrast, no significant differences were observed in left ventricular end‐diastolic diameter (LVEDD) (Figure 6D) or left ventricular end‐diastolic volume (LVEDV) (Figure 6G). These results highlight that the deficits in cardiac function of mdx‐Des^+/−^ mice primarily arise from altered cardiac contractility in systole, leading to a reduced blood ejection. The impairment of cardiac function in mdx‐Des^+/−^ mice compared to mdx and WT mice was further supported by qRT‐PCR analysis, which demonstrated an increased expression of the stress‐induced *Nppa*, *Nppb*, and *Myh7* genes in mdx‐Des^+/−^ compared to mdx and WT mice (Figure 6I). In contrast, no significant changes in fibrosis, revealed by qRT‐PCR, were observed in mdx‐Des^+/−^ compared to mdx mice (Figure 6J). Taken together, our findings show that restoration of WT levels of insoluble desmin in mdx hearts counteracts the advantage of a milder phenotype in mdx mice, in support of a protective role for desmin in DMD pathophysiology.

## 4 Discussion

In this work, we report that insoluble desmin increases in the cardiac tissue of mdx and D2.mdx mouse models of DMD, and this increase is associated with attenuated dystrophic phenotypes. Indeed, the canine model of DMD, the GRMD dog which develops a severe form of the disease [49], did not show such modulation in cardiac desmin levels. This observation suggests that endogenous insoluble desmin upregulation may exert a protective effect in the context of DMD. This hypothesis is further supported by our finding that mdx mice lacking both dystrophin and desmin (mdx‐Des^−/−^, dKO) presented an exacerbated cardiac phenotype, characterized by dilated cardiomyopathy according to standard criteria such as echocardiography data [50], elevated expression of cardiac stress markers (*Nppa*, *Nppb*, *Myh7*) [51, 52], and increased markers of myocardial fibrosis (*Col1a1, Col3a1, Ctgf, Vim*) [53]. In addition, in mdx‐Des^+/−^ mice, the reduction of insoluble desmin to levels comparable to WT hearts counteracts the advantage of a milder phenotype in mdx mice. The mdx‐Des^+/−^ mice have more pronounced systolic dysfunction, ventricular dilation, and adverse remodeling compared to mdx mice. These findings support the idea that desmin could act as a modifier of the cardiac phenotype in DMD by contributing to the maintenance of structural integrity of cardiomyocytes in the absence of dystrophin.

Increased insoluble desmin levels, clearly detected in mdx hearts via biochemical fractionation, did not result from pathological aggregates or inclusion bodies as in other cases [54]. Confocal immunofluorescence microscopy of mdx cardiac sections revealed only filamentous structures of desmin, reinforcing the hypothesis of a protective role of desmin filaments in the disease context. The upregulation of insoluble/filamentous desmin may represent an adaptive response to the chronic mechanical stress experienced by dystrophin‐deficient cardiomyocytes. DMD leads to a major disorganization of the cytoskeleton and disruption of extracellular matrix that are common among cardiac disorders leading to heart failure. Intermediate filaments such as desmin possess remarkable mechanical strain‐stiffening properties: they are compliant to small deformations and become more viscoelastic when large mechanical stress is applied so that they can be stretched up to 3.5‐fold without breaking [55]. These properties give desmin filaments a unique capacity to reinforce mechanical junctions, and to strengthen and stabilize the cell architecture under stress [34, 56]. Interestingly, this behavior is different from that of microtubules and actin microfilaments, which break when strain is increased. Although the precise role of desmin in cardiomyopathy progression remains only partially understood [34, 57], our results position this protein as a key structural player in the context of DMD.

The main identified trans‐acting modifiers in DMD concern genes related to inflammation and fibrosis, such as *SPP1*, *CD40*, *LTBP4*, *THSB1* [3]. Interestingly, the sarcomeric protein α‐actinin‐3, a constituent of Z‐lines of muscle fibers and an F‐actin cross‐linking protein, has also been identified as a modifier of DMD [27, 29]. In this register, it is not surprising that desmin, another Z‐line protein, can fulfill such a function. Modifiers have been identified mainly by a hypothesis‐driven candidate gene strategy or genome mapping/sequencing approach, such as genome‐wide association studies. Different DMD patients are genotyped for the single‐nucleotide polymorphism (SNP) of interest, and the phenotype is compared between genotype groups. In general, the SNPs could influence the expression levels of the gene of interest, which is involved in the development of DMD, or they could change the activity of the protein. In our study, a notable difference between WT and mdx mice is observed not at desmin expression levels but rather in the partition of soluble and insoluble desmin protein. Therefore, our results indicate that a modifier of DMD could play a role at the post‐translational level, expanding the field of modifier identification.

We hypothesized that the increase of insoluble desmin is due to an enhanced stabilization of filaments and looked for possible implicated factors. To this end, we investigated the post‐translational modifications of desmin which can be implicated in filament assembly and stability [43, 44], focusing on phosphorylation and *O*‐GlcNAcylation. Our analysis showed that only the insoluble fraction of desmin was modified in mdx hearts by increased phosphorylation. No significant changes in *O*‐GlcNAcylation were detected, suggesting a minor role in this context. Interestingly, our findings seem to imply that phosphorylation may contribute to the stabilization of desmin filaments, a hypothesis that contrasts with the widely accepted view that in muscle cells, this modification promotes filament disassembly [58]. However, a stabilizing effect of phosphorylation has been observed in neurofilaments, which can be hyperphosphorylated without depolymerizing, particularly in postmitotic neurons [59]. Therefore, the role of phosphorylation in filament dynamics may be highly context‐dependent. It is possible that, in the particular context of dystrophin deficiency, presently unknown phosphorylated sites on desmin have positive effects on filament assembly and/or stability, an interesting hypothesis to be addressed in future detailed studies.

Furthermore, we explored the expression of several proteins potentially involved in the regulation of desmin filament stability: calpain‐1, a protease known to mediate desmin degradation [60, 61, 62]; αB‐crystallin and HSP27, two small heat shock proteins that promote desmin stabilization [44, 63, 64]; and BAG3, a co‐chaperone involved in chaperone‐assisted selective autophagy (CASA) [65]. The observed decrease in calpain‐1 in mdx hearts could slow the degradation of desmin, favoring the stability of the filament. Besides, in the soluble desmin fractions, the bands with molecular weights lower than 53 kDa of full desmin protein (Figures 1B and 3B,D) could be the product of calpain‐1 digestion and, interestingly, appear to be weaker in mdx cardiac tissue (Figures 1B and 3B) with lower levels of calpain‐1 protein. On the other hand, αB‐crystallin and HSP27 protein levels were both increased in mdx hearts. These small heat shock proteins act as molecular chaperones that assist in the folding and stabilization of intermediate filaments under mechanical or oxidative stress. Critical αB‐crystallin‐binding sites have been identified in the tail domain of desmin [66]. It has been shown that binding to αB‐crystallin during filament assembly can influence the topology of formed desmin filaments and potentially modulate their interaction with other sarcomeric partners. In addition, the activation of HSP27 in cardiomyocytes under stress conditions protected desmin from calpain‐mediated proteolysis, indicating a complementary role in filament stabilization [61, 67].

We propose that the decrease in calpain‐1, together with the upregulation of both αB‐crystallin and HSP27, represents a coordinated mechanism supporting desmin filament accumulation by combining reduced proteolytic degradation with enhanced chaperone‐mediated stabilization. Our results also show a significant increase in BAG3 expression, which may reflect the activation of the CASA pathway in mdx cardiomyocytes, with the aim of preventing aggregation of upregulated proteins accumulating within the cytoplasm, including desmin. This could contribute to the lack of desmin aggregates despite the increase of insoluble protein in mdx hearts.

To date, increased desmin in the context of dystrophin deficiency has been observed only in mice. It would be interesting to determine whether the differences observed between mice and dogs are primarily driven by species‐specific mechanisms of physiological adaptation. Of course, the ultimate objective for future studies will be to define the state of desmin filaments in DMD patients. Could an increase in insoluble/filamentous desmin in patient striated muscles result in a milder phenotype, offering therapeutic opportunities? Different drug‐based and molecular therapeutic approaches have been proposed for the treatment of DMD [68]. Among those, the upregulation of utrophin, a genetic and functional paralogue of dystrophin, has been extensively investigated as a therapeutic option and could serve as a paradigm for desmin. Utrophin is upregulated in mdx mice and appears to partially compensate for the dystrophin loss in patients [69]. Investigated strategies to upregulate utrophin include mRNA and protein stabilization [70, 71], genome editing [72, 73], and recently, nonsense‐mediated dystrophin mRNA decay [69]. By analogy, similar strategies could be applied to find the drugs and/or molecular therapeutic approaches for the upregulation of desmin. Besides, the upregulation of both utrophin and desmin in DMD patients could be an interesting therapeutic option to exploit. Further studies will also be required to elucidate the precise functional relationships between desmin, its post‐translational modifications, and regulatory partners, in order to understand the mechanisms of filament stabilization in the dystrophic mouse heart and find a therapeutic strategy for DMD.

## Author Contributions

**Brice‐Emmanuel Guennec:** data curation, formal analysis, investigation, methodology, validation, visualization, writing – original draft. **Yeranuhi Hovhannisyan:** data curation, formal analysis, investigation, methodology, validation, visualization, writing – review and editing. **Gaëlle Revet:** data curation, formal analysis, investigation, methodology. **Sila Polat:** data curation, formal analysis, investigation, methodology. **Medhi Hassani:** data curation, formal analysis, investigation, methodology. **Nathalie Mougenot:** data curation, formal analysis, investigation, methodology. **Inès Barthelemy:** investigation, formal analysis, validation, writing – review and editing. **Stephane Blot:** formal analysis, validation, writing – review and editing. **Caroline Cieniewski‐Bernard:** formal analysis, investigation, methodology, validation, writing – review and editing. **Arnaud Ferry:** formal analysis, investigation, methodology, validation, writing – review and editing. **Ekaterini Kordeli:** conceptualization, data curation, formal analysis, investigation, methodology, resources, supervision, validation, visualization, writing – original draft. **Zhenlin Li:** conceptualization, data curation, formal analysis, funding acquisition, investigation, methodology, project administration, resources, supervision, validation, visualization, writing – original draft. **Onnik Agbulut:** conceptualization, data curation, formal analysis, funding acquisition, investigation, methodology, project administration, resources, supervision, validation, visualization, writing – original draft.

## Conflicts of Interest

The authors declare no conflicts of interest.

## Supporting information


**Figures S1–S7:** apha70117‐sup‐0001‐FigureS1‐S7.docx.

## Data Availability

The data that support the findings of this study are available from the corresponding author upon reasonable request.
