# Supplementary material for: Exploring Desmin as a Potential Modifier in Duchenne Muscular Dystrophy–Associated Cardiomyopathy
Source: Acta Physiol (Oxf). 2025 Oct 29;241(12):e70117. doi: 10.1111/apha.70117 (PMC12572696; doi:10.1111/apha.70117)
Supplement: Supplementary file 1 — Figures S1–S7: apha70117‐sup‐0001‐FigureS1‐S7.docx. [file APHA-241-e70117-s001.docx]

Supplementary Information

**Exploring Desmin as a Potential Modifier in Duchenne Muscular Dystrophy-associated Cardiomyopathy**

Brice-Emmanuel Guennec^1^, Yeranuhi Hovhannisyan^1^, Gaëlle Revet^1^, Sila Polat^1^, Medhi Hassani^1^, Nathalie Mougenot^2^, Inès Barthelemy^3^, Stephane Blot^3^, Caroline Cieniewski-Bernard^4^, Arnaud Ferry^5,6^, Ekaterini Kordeli^1^, Zhenlin Li^1^, Onnik Agbulut^1^*

^1^Sorbonne Université, Institut de Biologie Paris-Seine (IBPS), UMR CNRS 8263, INSERM U1345, Development, Adaptation and Ageing, Paris-France.

^2^Sorbonne Université, UMS28, Plateforme d’Expérimentation Cœur, Muscles, Vaisseaux, Paris-France.

^3^Inserm U955-E10, IMRB, Université Paris Est, Ecole nationale vétérinaire d'Alfort, 94700, Maisons-Alfort-France.

^4^Université de Lille, CNRS, UMR 8576 - UGSF - Unité de Glycobiologie Structurale et Fonctionnelle, Lille-France.

^5^ Sorbonne Université, Centre de Recherche en Myologie, UMRS974, Paris-France.

^6^ Université Paris Cité,Paris-France.

**Short title: Desmin as a Modifier in DMD Cardiomyopathy**

*** Corresponding author**

Prof OnnikAgbulut, Institut de Biologie Paris-Seine, UMR CNRS 8263, INSERM U1345, 7, quai St Bernard (case 256), 75005 Paris-France. Email : [onnik.agbulut@sorbonne-universite.fr](mailto:onnik.agbulut@sorbonne-universite.fr)

Dr Zhenlin Li, Institut de Biologie Paris-Seine, UMR CNRS 8263, INSERM U1345, 7, quai St Bernard (case 256), 75005 Paris-France. Email : [zhenlin.li@sorbonne-universite.fr](mailto:onnik.agbulut@sorbonne-universite.fr)

**
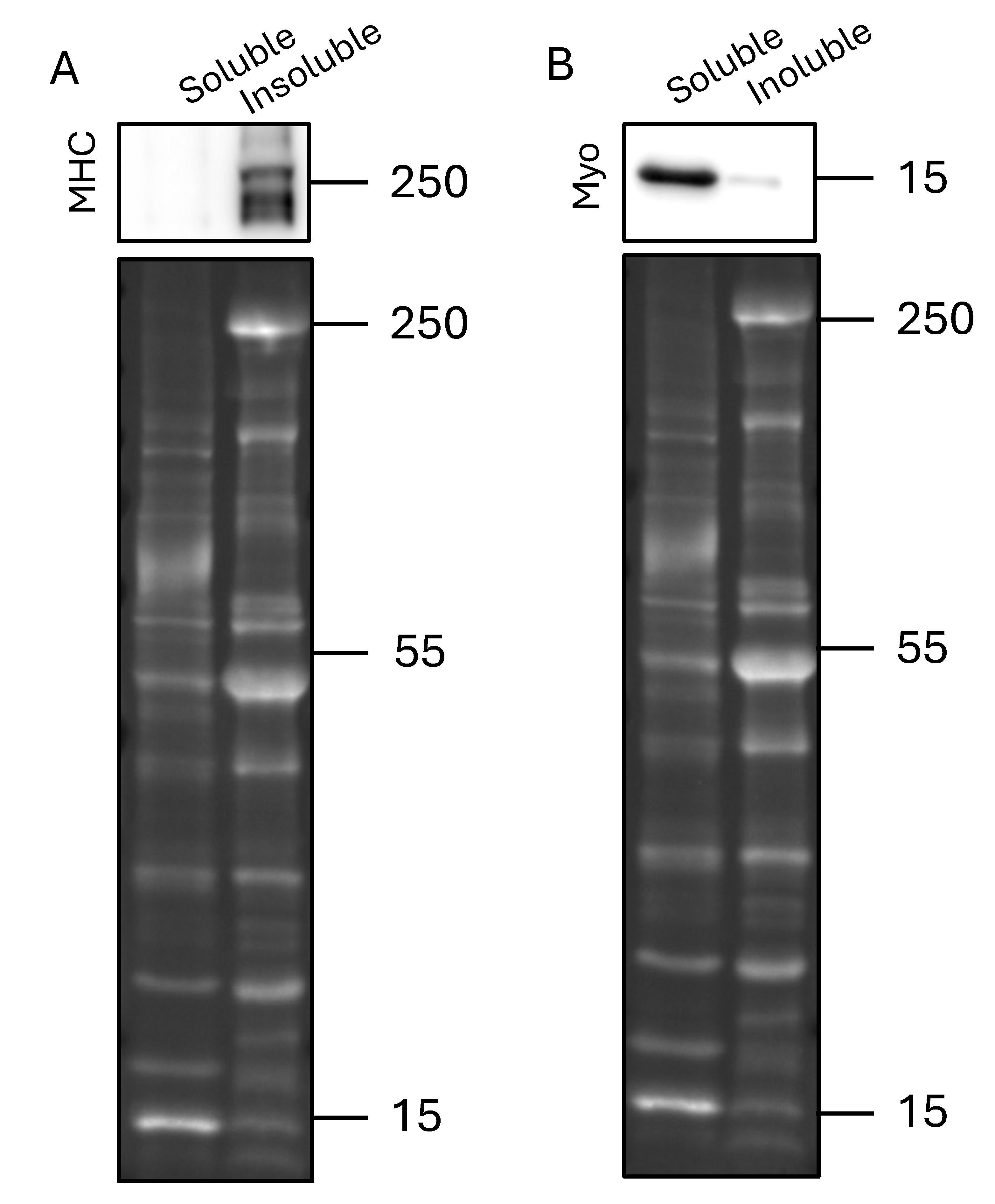
**

**Figure S1. Purity of isolated protein fractions extracted from hearts of 4-month-old male mice**. Representative Western blots of **(A)** Myosin Heavy Chain (MHC), an insoluble protein marker, and **(B)** Myoglobin (Myo), a soluble protein marker, and corresponding total protein membranes (No Stain).

**
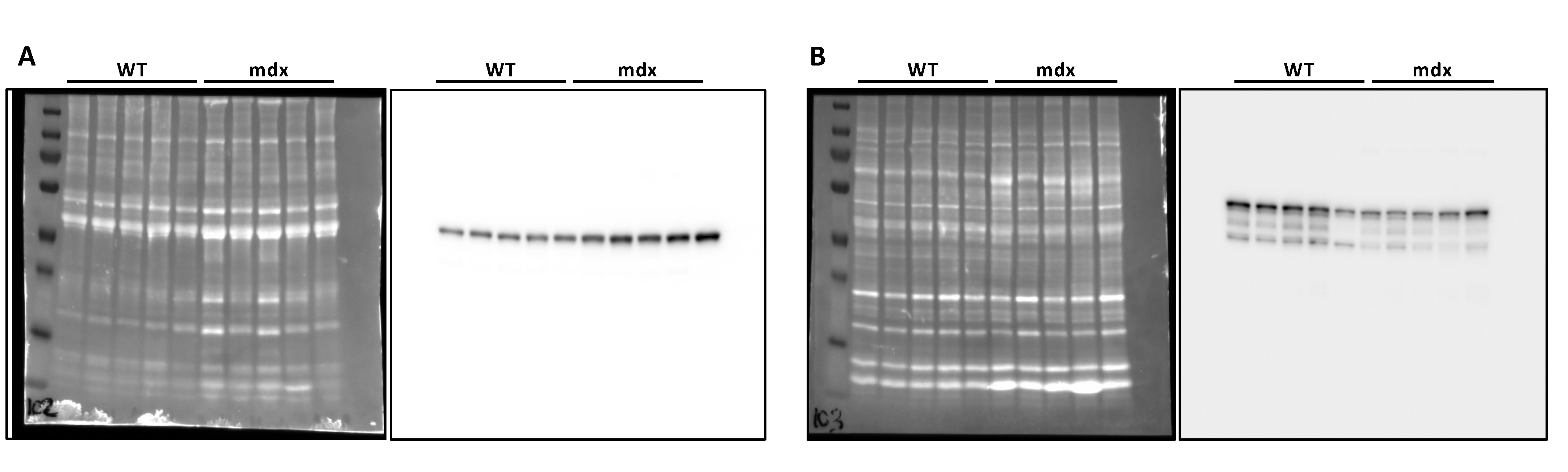
**

**Figure S2.** **Protein membranes used in Figure 1 for quantification in 4-month-old male mdx mice.** Total protein membranes (No-Stain) and corresponding Western blot membranes used for protein quantification. Panels show **(A)** desmin insoluble fraction and **(B)** desmin soluble fraction.

**Figure S3. Protein membranes used in Figure 2 for quantification in 4-month-old male mdx mice.** Total protein membranes (No-Stain) and corresponding Western blot membranes used for protein quantification. Panels show: **(A)** Phos-tag PAGE of the desmin insoluble fraction, **(B)** Phos-tag PAGE of the desmin soluble fraction, **(C)** WGA-PAGE of the desmin insoluble fraction, **(D)** WGA-PAGE of the desmin soluble fraction, **(E)** calpain-1, **(F)** αB-crystallin, **(G)** BAG3 (upper) and HSP-27 (lower). For panels (E, F and G), membranes were cut prior to antibody incubation.


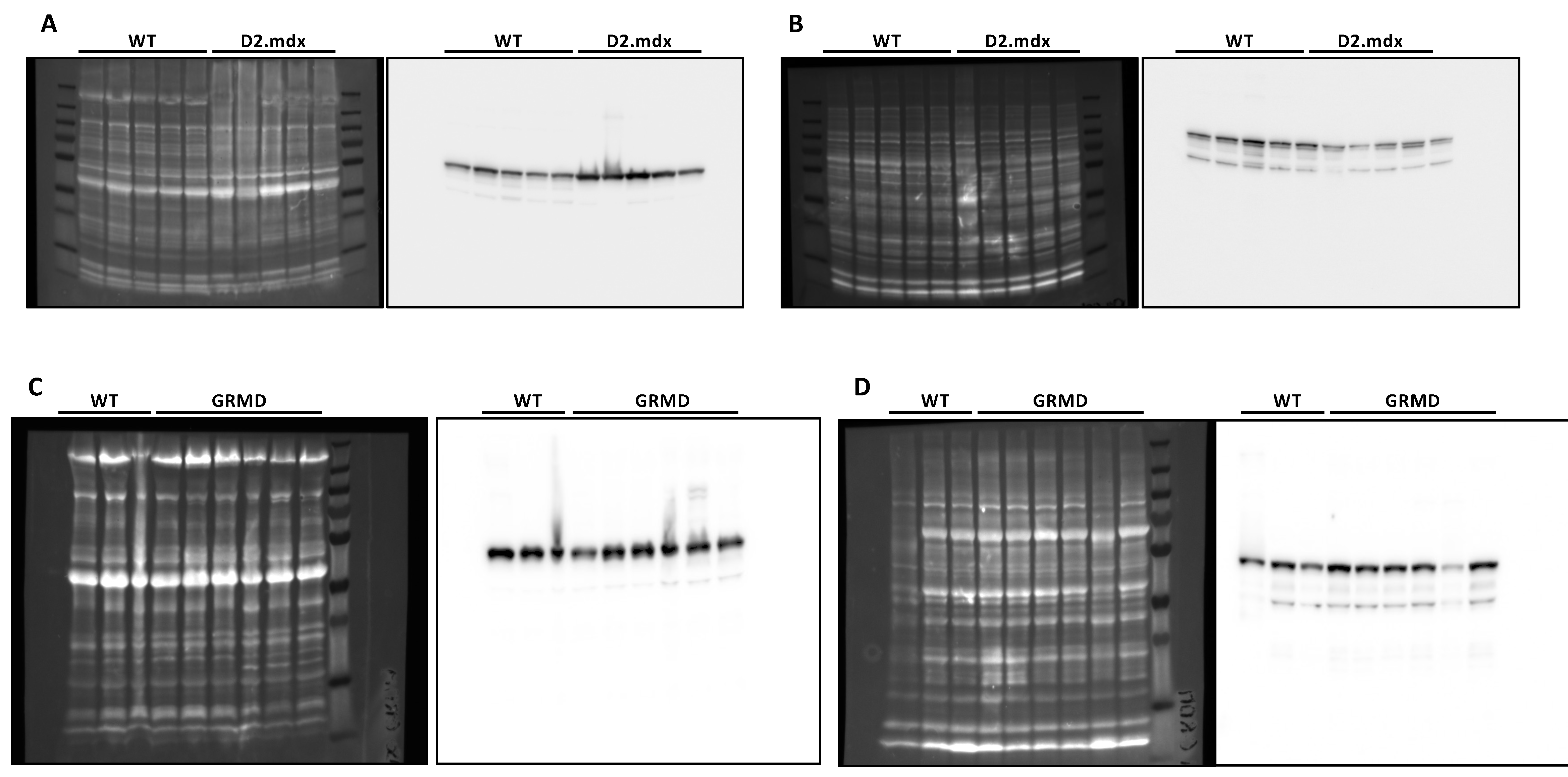


**Figure S4. Protein membranes used in Figure 3 for quantification in 4-month-old male D2.mdx mice and GRMD dogs.** Total protein membranes (No-Stain) and corresponding Western blot membranes used for protein quantification. Panels show: **(A)** desmin insoluble fraction and **(B)** desmin soluble fraction in D2.mdx mice; **(C)** desmin insoluble fraction and **(D)** desmin soluble fraction in GRMD dogs.

**
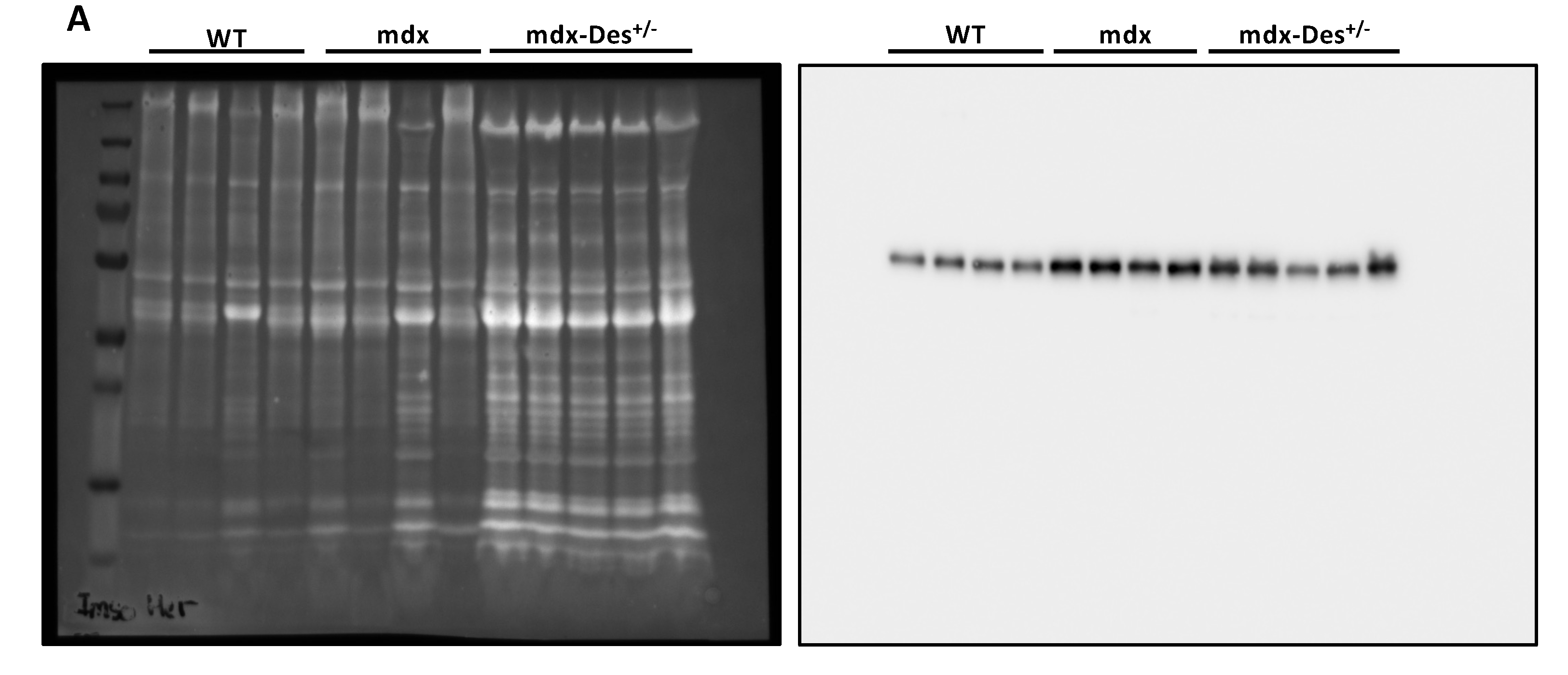
**

**Figure S5. Protein membranes used in Figure 6 for quantification in 4-month-old male mdx-Des⁺/⁻ mice.** Total protein membranes (No-Stain) and corresponding Western blot membranes used for protein quantification. Panel shows desmin insoluble fraction.

**Figure S6. Total desmin protein levels in mdx and WT mice.** Western blots and corresponding densitometric analyses of total desmin levels extracted from hearts of 4-month-old male WT and mdx mice (n=5 per group). Protein levels were normalized to the total protein band profile (No-Stain) per lane and are expressed as mean values ± SEM.

**Figure S7. Results from two-step normalization of Phos-tag PAGE and WGA-PAGE from desmin insoluble and soluble fractions from 4-month-old male WT and mdx mice.** For each sample, results were first normalized to the total protein loading **(A, C)**, (results of **Figure 2**) and subsequently referred to total desmin levels **(B, D)** from the corresponding insoluble (results of **Figure 1A**) or soluble (results of **Figure 1B**) fractions, previously normalized to total protein loading as well. These two-step normalization of phosphorylation **(B)** and *O*-GlcNAcylation **(D)** results take in account the modifications of total mdx desmin levels in soluble and insoluble fractions. Results are expressed as mean values ± SEM. *, *p* < 0.05, **, *p* < 0.01, ****, *p* < 0.0001.
